# Twist expression promotes migration and invasion in hepatocellular carcinoma

**DOI:** 10.1186/1471-2407-9-240

**Published:** 2009-07-18

**Authors:** Noriyuki Matsuo, Hidenori Shiraha, Tatsuya Fujikawa, Nobuyuki Takaoka, Naoki Ueda, Shigetomi Tanaka, Shinichi Nishina, Yutaka Nakanishi, Masayuki Uemura, Akinobu Takaki, Shinichiro Nakamura, Yoshiyuki Kobayashi, Kazuhiro Nouso, Takahito Yagi, Kazuhide Yamamoto

**Affiliations:** 1 Department of Gastroenterology and Hepatology, Okayama University Graduate School of Medicine and Dentistry, 2-5-1 Shikata-cho, Okayama 700-8558, Japan; 2 Department of Gastroenterological Surgery, Transplant, and Surgical Oncology, Okayama University Graduate School of Medicine and Dentistry, 2-5-1 Shikata-cho, Okayama 700-8558, Japan

## Abstract

**Background:**

Twist, a transcription factor of the basic helix-loop-helix class, is reported to regulate cancer metastasis. It is known to induce epithelial-mesenchymal transition (EMT). In this study, we evaluated the expression of twist and its effect on cell migration in hepatocellular carcinoma (HCC).

**Methods:**

We examined twist expression using immunohistochemistry in 20 tissue samples of hepatocellular carcinoma, and assessed twist expression in HCC cell lines by RT-PCR and Western blot analysis. Ectopic twist expression was created by introducing a twist construct in the twist-negative HCC cell lines. Endogenous twist expression was blocked by twist siRNA in the twist-positive HCC cell lines. We studied EMT related markers, E-cadherin, Vimentin, and N-cadherin by Western blot analysis. Cell proliferation was measured by MTT assay, and cell migration was measured by *in vitro *wound healing assay. We used immunofluorescent vinculin staining to visualize focal adhesion.

**Results:**

We detected strong and intermediate twist expression in 7 of 20 tumor samples, and no significant twist expression was found in the tumor-free resection margins. In addition, we detected twist expression in HLE, HLF, and SK-Hep1 cells, but not in PLC/RPF/5, HepG2, and Huh7 cells. Ectopic twist-expressing cells demonstrated enhanced cell motility, but twist expression did not affect cell proliferation. Twist expression induced epithelial-mesenchymal transition together with related morphologic changes. Focal adhesion contact was reduced significantly in ectopic twist-expressing cells. Twist-siRNA-treated HLE, HLF, and SK-Hep1 cells demonstrated a reduction in cell migration by 50, 40 and 18%, respectively.

**Conclusion:**

Twist induces migratory effect on hepatocellular carcinoma by causing epithelial-mesenchymal transition.

## Background

Hepatocellular carcinoma (HCC) is one of the most fatal cancers, especially in eastern Asia [1-3]. Despite the recent advances in diagnosis and treatment of HCC, the mortality rate of HCC remains high [2,3]. Many resources have been devoted to prevention through better understanding of the causes and finding cures for HCC. Viral infections that cause chronic hepatitis and progressive liver cirrhosis are known to cause HCC [1,4]. Although new therapies for chronic hepatitis have been developed, the number of HCC patients has not declined [5,6].

The treatment of HCC is dependent on the tumor stage at the time of diagnosis. Potentially curative hepatic resection and partial ablation therapy are reserved for patients in the earlier stages of HCC [7,8]. A major reason for poor prognosis of HCC is the recurrence and metastasis after surgery or ablation therapy. Therefore, prevention of metastasis is important for HCC therapy [9].

Metastasis is a complex process and various factors are involved in each step of metastasis [10]. Recent studies suggest that numerous genes and proteins involved in essential roles during embryonic development are mutated or aberrantly expressed in different cancers [11-13]. Epithelial-mesenchymal transition (EMT) is a characteristic of the most aggressive metastatic cancer cells [14-19]. Cells that undergo EMT morphogenesis switch from an apical-basolateral, polarized epithelial phenotype to a spindle-shaped, fibroblast-like mesenchymal phenotype. In their natural state, epithelial cells exist as tight cell clusters that maintain cell-cell or cell-to-matrix contacts, whereas mesenchymal cells are loosely organized, unpolarized cells with reduced adhesion and enhanced migratory tendencies. A key feature in the initiation and execution of EMT is the down regulation of E-cadherin expression [14-16]. Several mechanisms that down-regulate E-cadherin expression have been reported recently [20-22].

A transcriptional factor involved in down-regulation of E-cadherin, twist, has been shown to play a crucial role in carcinoma metastasis. Microarray analysis revealed that twist is predominantly expressed in metastatic cancers [23-25]. Furthermore, a loss of twist expression prevents the intravasation of metastatic tumor cells into the blood circulation [25]. Twist-enhanced cancer metastasis includes breast cancer, gastric cancer, and HCC [24,26,27].

Therefore, we conducted the present study to investigate the role of twist in cell migration of HCC cells and its role in metastasis.

## Methods

### HCC tissues and immunohistochemistry

Twenty patients including 12 men with ages ranging from 53 to 77 years (average age, 64 years) and eight women with ages ranging from 54 to 82 years (average age, 64 years) at the time of hepatic resection were included in this study. HCC tissues along with adjacent liver tissues were used for analysis. We obtained informed consent from all donors of liver tissue samples, as per the institutional guidelines, and the study was approved by the Research Ethics Committee of Okayama University.

We performed immnunohistochemistry on formalin-fixed paraffin sections. The sections were dewaxed and dehydrated. After rehydration, endogenous peroxidase activity was blocked for 30 min using a methanol solution containing 0.3% hydrogen peroxide. After antigen retrieval in citrate buffer, we blocked the sections overnight at 4°C. The sections were probed with goat polyclonal antibody (Santa Cruz Biotechnology Inc., Santa Cruz, CA, USA). The primary antibody was detected using a biotinylated anti-goat antibody (Dako Japan, Tokyo, Japan). The signal was amplified by avidin-biotin complex formation and developed using diaminobenzidine followed by counterstaining with hematoxylin. Next, the samples were dehydrated in alcohol and xylene and mounted onto glass slides. The sections were scored for twist expression using a four-titer scale: 0, negative; 1, weak signal; 2, intermediate signal; and 3, strong signal [28]. Two observers, who were blinded to the samples, independently scored the sections. We reviewed all discrepancies in the scoring process and reached a consensus.

### Cell lines and cell culture

We obtained the human HCC cell lines, HLE, HLF, and Huh7 from the Health Science Research Resources Bank (Osaka, Japan), and SK-Hep1, PLC/PRF/5 (PLC), and HepG2 cell lines from the American Type Culture Collection (Manassas, VA, USA). We maintained the cells in Dulbecco's modified Eagle's medium (Invitrogen, Carlsbad, CA, USA). Media were supplemented with 10% heat-inactivated fetal bovine serum [(FBS), Sigma, St. Louis, MO, USA], 1% nonessential amino acid (Sigma), 1% sodium pyruvate (Sigma), and 1% penicillin/streptomycin solution (Sigma). We cultured the cells at 37°C in a humidified atmosphere of 5% CO_2 _and 95% air. Cells were quiesced using restricted serum conditions of 0.1% dialyzed FBS at subconfluence for 24 h prior to the experiments.

### Determination of twist expression levels in HCC cell lines

We determine twist expression levels in HCC cell lines by reverse transcriptase-polymerase chain reaction (RT-PCR). Total RNA was isolated from HCC cell lines using TRIzol™ reagent (Invitrogen). Reverse transcription was performed with extracted RNA using random primers and RiverTra Ace™ (Toyobo, Osaka, Japan) reverse transcriptase. Sense oligonucleotide primer (5'-CGCCCCGCTCTTCTCCTCT-3') and antisense primer (5'-GACTGTCCATTTTCTCCTTCTCTG-3') were designed using a primer select program in the Lasergene™ software (DNAStar, Madison, WI, USA) based on the published sequence of twist (Gene Bank accession no. NM000474). All PCR reactions were performed under the following conditions. The reaction mixture consisted of 100 ng template DNA, 3.2 pmol forward primer, 3.2 pmol reverse primer, 0.4 μL deoxynucleoside triphosphates (25 mM each), 2.5 U pfu turbo DNA polymerase (Stratagene, LaJolla, CA, USA), and 5.0 μL 10× pfu reaction buffer. The volume of the reaction mixture was adjusted to 50 μL with sterile distilled H_2_O. PCR amplification was performed in an iCycler™ (Bio-Rad, Hercules, CA, USA) using the following conditions: cycle 1, 95°C for 2 min; cycle 2–30, 95°C for 30 s, 56°C for 30 s, and 72°C for 90 s and a final elongation step of 72°C for 10 min.

### Immunoblot analysis

We grew the cells to confluence in 6-well tissue culture plastic dishes. The cells were washed two times with cold PBS, lysed with 150 μL of sample buffer (100 mM Tris-HCl pH6.8, 10% glycerol, 4% sodium dodecyl sulfate [SDS], 1% bromophenol blue, 10% β-mercaptoethanol), resolved by SDS-polyacrylamide gel electrophoresis (PAGE), and finally transferred to an Immobilon-P™ polyvinylidene difluoride membrane (Millipore Corporation, Bedford, MA, USA). We blocked the membranes using Tris-buffered saline with Tween 20 (Sigma) (TBS-T) buffer containing 1% bovine serum albumin for 1 h. Next, we incubated the membranes with anti-twist antibody (Santa Cruz Biotechnology Inc.), anti-E-cadherin antibody (Cell Signaling Technology, Denvers, MA, USA), anti-vimentin antibody (Santa Cruz Biotechnology Inc.), anti-N-cadherin antibody (Cell Signaling Technology), and anti-α-actin antibody (Sigma). We washed the membranes three times with TBS-T and probed with horseradish peroxidase-conjugated secondary antibody before developing them using an ECL Western blotting detection system (Amersham Biosciences, Piscataway, NJ, USA) by enhanced chemiluminescence.

### Growth proliferation assay

Cell proliferation was assessed by 3-(4,5-dimethylthiazol-2-yl)-2,5-diphenyl tetrazolium bromide (MTT) assay. In brief, we plated the cells in 96-well tissue culture plastic dishes at a concentration of 10^4 ^cells/mL. After 24 h of quiescence, the cells were cultured for 72 h with or without 10% FBS. At the end of the treatment, 10 μL MTT (5 mg/mL in phosphate buffered saline) was added to each well and incubated for an additional 4 h at 37°C. The colored formazan product was then dissolved in 100 μL of DMSO. We evaluated the activity of the mitochondria, reflecting cellular growth and viability, by measuring the optical density at 570 nm using a microplate reader (BioRad).

### Cell migration assay

We assessed cell migration by determining the ability of the cells to move into an acellular space in a two-dimensional *in vitro *"wound healing assay" [29]. In brief, cells were grown to confluence in 6-well tissue culture plastic dishes to a density of approximately 5 × 10^6 ^cells/well. After 24 h of quiescence, the cells were denuded by dragging a rubber policeman (Fisher Scientific, Hampton, NH, USA) through the center of the plate. Cultures were rinsed with PBS and replaced with fresh quiescent medium alone or containing 10% FBS, following which the cells were incubated at 37°C for 24 h. Photographs were taken at 0 and 24 h, and the relative distance traveled by the cells at the acellular front was measured.

### Cloning and transfection of Twist

We obtained human twist cDNA by PCR-based cloning from a normal human placental cDNA library (Takara Bio Inc., Otsu, Japan). In brief, cDNA was amplified by PCR using sense and antisense primers and Pfu Turbo DNA polymerase (Stratagene) and cloned into PCR II TA cloning vector (Invitrogen). The size of the PCR product was ~750 bp. After confirming the sequence, the twist cDNA was subcloned into pcDNA 3.1 expression vector (Invitrogen) downstream from a cytomegalovirus (CMV) promoter. Twist and/or CAT (control) constructs were transfected into the HCC cell lines using Superfect™ transfection reagent (Qiagen, Hilden, Germany), as per the manufacture's instructions. After transfection, the cells were incubated for 24–48 h and utilized for the following experiments.

### Morphological analysis and immunofluorescence staining

We plated cells in 2 mL media in 6-well tissue culture plastic dishes at a concentration of 10^5 ^cells/mL. After 48 h of transfection, we observed the morphology under a phase-contrast microscope. For immunofluorescence staining, cells were seeded on Lab-Tek™ slide chamber (Nunc Rochester, NY, USA). After 24 h incubation, cells were fixed for 20 min in 3% freshly hydrolyzed paraformaldehyde in PBS at room temperature. Cells were first stained with rhodamine-phaloidin (Invitrogen) and then with an anti-vinculin antibody (Sigma) for 30 min at room temperature, washed three times with PBS, and visualized using Oregon Green conjugated secondary antibody (Invitrogen). Photographs were taken using a fluorescence microscope (BX51, Olympus, Tokyo, Japan) equipped with a digital image capturing system (DP50, Olympus). The numbers of focal adhesion complexes were determined by vinculin staining.

### Gene silencing with small interfering RNA (siRNA)

We obtained siRNA duplexes targeting twist sequences and control (Silencer Negative™) from Applied Biosystems (Foster City, CA, USA). We transfected the siRNAs into the cells using RNAiFect™ transfection reagent (Qiagen), and incubated the cells for 48 h before the analysis. The interference of twist protein expression was confirmed by immunoblot analysis using anti-twist antibody. Cell migration assay was performed, as described above.

## Results

### Twist-expression in HCC tissues

**Figure 1:**
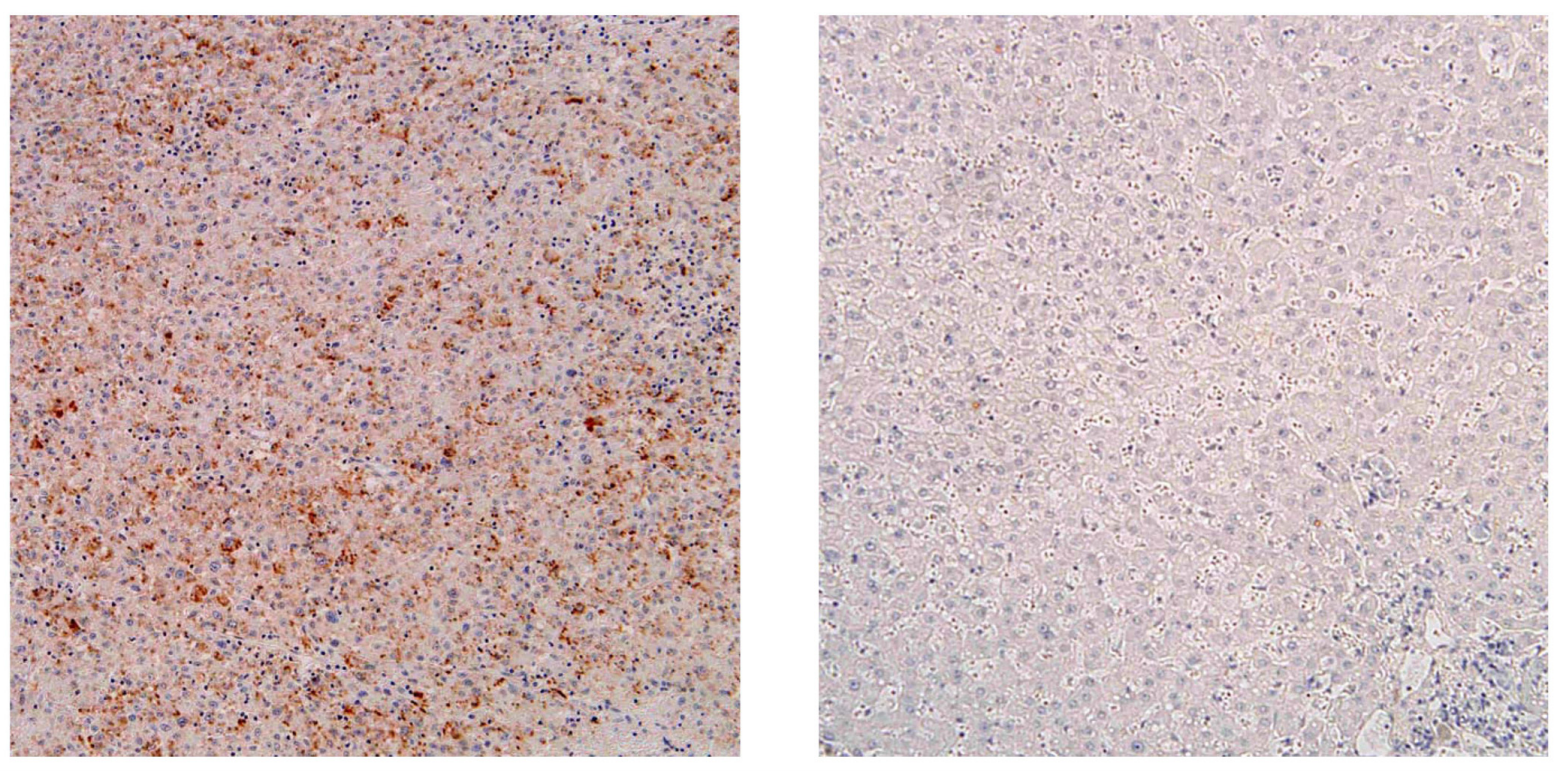
**Twist expression in HCC tissue and its adjacent liver tissue**. Twist expression was assessed by immunohistochemical staining in a human HCC tissue (left panel) and corresponding tumor-free section (right panel). Photographs were taken using a light microscope (BX51, Olympus) equipped with a digital image capturing system (DP50, Olympus). Shown are the representative images of twist expressing a HCC tissue and its corresponding tumor-free section.

Immunohistochemical analysis revealed twist expression in both cytoplasm and nuclei in HCC tissues with enhanced expression in the cytoplasm in most cases. Representative images of twist-positive HCC and the corresponding tumor-free section are shown in Figure 1. Strong (Score 3) and intermediate (Score 2) twist expression was detected in 7 of 20 tumor samples (Figure 1 and Table 1), while no (Score 0) significant twist expression was found in tumor-free resection margins. We observed no significant difference in clinicopathologic features in high twist expressing samples compared with low twist expressing samples (Table 1).

**Table 1:** Twist expression levels and clinicopathologic features.

Twist expression score	0 (n = 7)	1 (n = 6)	2 (n = 6)	3 (n = 1)
***pTNM stage***				
Stage I	1	0	1	1
Stage II	2	2	4	0
Stage IIIA	4	4	1	0
Stage IIIB	0	0	0	0
Stage IV	0	0	0	0
***Distant metastasis***				
M0	7	6	6	1
M1	0	0	0	0
***Portal vein invasion***				
absence	7	3	4	1
presence	3	3	2	0

### Twist expression in HCC cell lines correlated cell character

**Figure 2:**
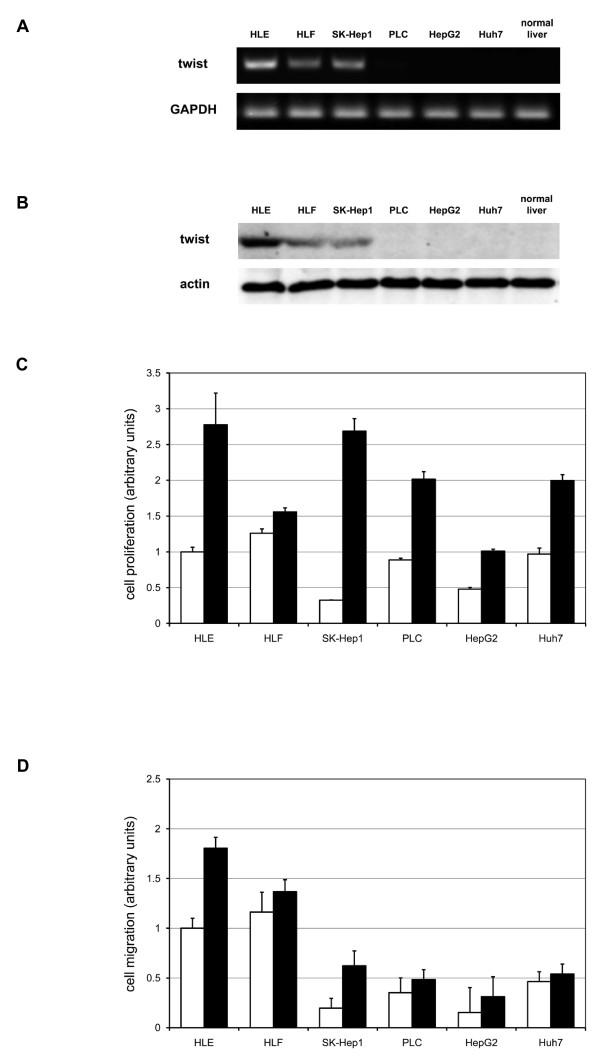
**Twist expression in HCC cell lines**. A, B. Cells were grown to confluence in 6-well tissue culture plastic dishes. Total RNA was extracted from various HCC cell lines. mRNA expression of twist and GAPDH were determined by RT-PCR using a twist-specific primer set (A). Shown are representative gel pictures of three independent studies. Twist protein expression was analyzed by SDS-PAGE and immunoblot using anti-twist antibody (B). Immunoblotting for α-actin expression levels was used to verify equal loading of cellular protein. Normal liver samples were used to determine twist expression level in nonmalignant tissue. Shown are representative blots of more than three independent studies. C, D. Cells were plated and quiesced for 24 h in DME with 0.1% dialyzed FBS. Later the cells were cultured with (black bars) or without (clear bars) 10% FBS. Cell proliferation (C) and cell migration activities (D) were assessed as described in Materials and Method. All results are expressed as a ratio to non-treated HLE cells. The data are the mean ± SEM of at least three independent studies each performed in triplicate.

To evaluate twist expression in various HCC cell lines, we assessed twist mRNA and protein expressions by RT-PCR and immnunoblot analysis, respectively (Figures 2A, B). Twist mRNA was expressed in HLE, HLF, and SK-Hep1 cells, while no detectable twist mRNA was found in PLC, HepG2, and Huh7 cells.

We assessed cell proliferation and migration activities in the HCC cell lines. Average basal cell proliferative activities of twist-positive and twist-negative HCC cell lines were 0.86 ± 0.04 and 0.78 ± 0.04, respectively (Figure 2C). FBS-induced cell proliferative activities of twist-positive and twist-negative HCC cell lines were 2.34 ± 0.22 and 1.67 ± 0.07, respectively (Figure 2C). Average basal cell migration activities of twist-positive and twist-negative HCC cell lines were 0.79 ± 0.08 and 0.32 ± 0.03, respectively (Figure 2D). FBS-induced cell migration activities of twist-positive HCC cell lines and twist-negative HCC cell lines were 1.26 ± 0.08 and 0.45 ± 0.02, respectively (Figure 2D).

### Twist expression did not stimulate cell proliferation in HCC cell lines

**Figure 3:**
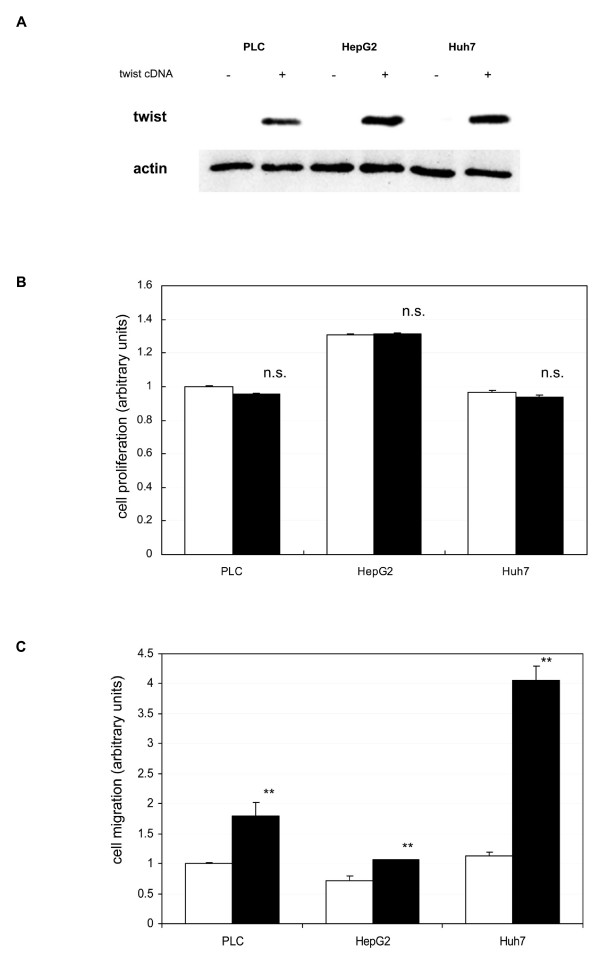
**Effect of ectopic twist expression**. CMV-driven eukaryotic expression constructs for CAT (control) and twist were introduced into PLC, HepG2, and Huh7 cells using Superfect™ chemical transfection reagent. A. After a 48-h incubation period, cells were lysed. Equal amounts of protein were analyzed by SDS-PAGE and immunoblotted with anti-twist and anti-α-actin antibodies. Shown are representative blots of more than three independent studies. B, C. After a 24-h incubation period, cells were quiesced for 24 h in DME with 0.1% dialyzed FBS. Then cells were cultured with 10% FBS. Cell proliferation (B) and cell migration activities (C) were assessed as described in Materials and Method. All results are expressed as a ratio to control CAT cDNA transfected PLC (CAT; clear bars, twist; black bars). The data are the mean ± SEM of at least three independent studies each performed in triplicate. Statistical analysis was performed by Student's *t*-test: **P < 0.01; n.s., not significant (vs CAT cDNA transfected cells).

To investigate the effect of ectopic twist expression, we introduced a twist construct into twist-negative HCC cell lines. As shown in Figure 3A, introduction of a twist construct induced expression of the twist protein. Cell growth proliferation was assessed by MTT assay (Figure 3B). In all three twist-negative HCC cell lines, twist expression did not have any effect on cell proliferation.

### Twist expression enhanced cell migration in HCC cell lines

To examine the effect of ectopic twist expression on HCC cell lines, we performed an *in vitro *wound healing assay. This assay is commonly used for assessing the effect of pro- and anti-migratory agents on cultured cells [30]. Twist expression significantly enhanced cell migration of HCC cell lines (Figure 3C). The twist-induced cell migration activities were 1.8-, 1.5-, and 3.6-fold in PLC, HepG2, and Huh7 cells, respectively.

### Induction of twist expression promotes EMT in HCC cell lines

**Figure 4:**
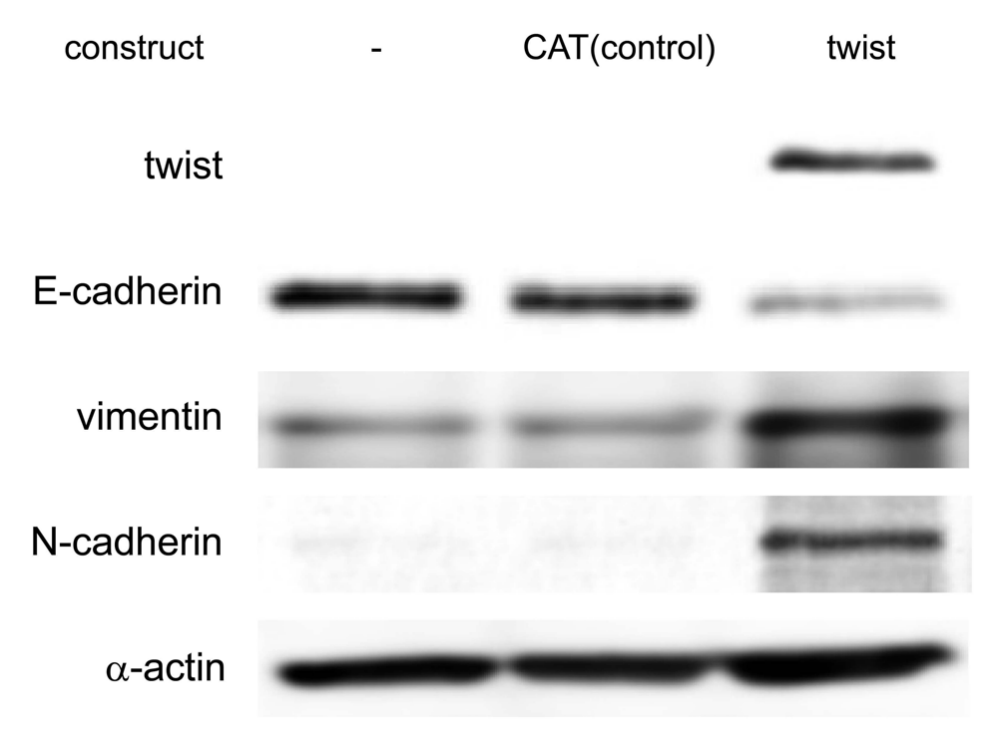
**EMT marker in twist expressed cell**. CMV-driven eukaryotic expression constructs for CAT (control) and twist were introduced into PLC cells using Superfect™ chemical transfection reagent. After a 48-h incubation period, cells were lysed. Equal amounts of protein were analyzed by SDS-PAGE and immunoblotted with anti-twist, anti-E-cadherin, anti-vimentin, anti-N-cadherin, and anti-α-actin antibodies. Shown are representative blots of more than three independent studies.

To investigate the effect of twist expression, we assessed the expression of EMT markers by immunoblot analysis (Figure 4). The expression of the epithelial marker, E-cadherin, was down-regulated, while the expression of mesenchymal markers, vimentin and N-cadherin, were up-regulated in twist-induced cells.

### Twist expression induces morphological change and reduces focal adhesion in HCC cell lines

**Figure 5:**
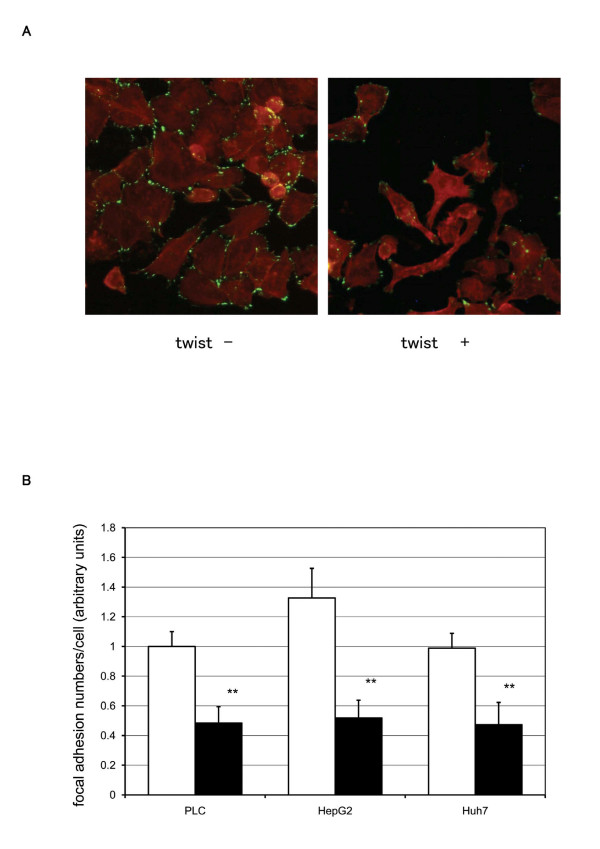
**Focal adhesion staining of twist expressed cells**. CAT (control) and twist cDNAs were introduced in PLC, HepG2, and Huh7 cells using Superfect™ chemical transfection reagent. After a 48-h incubation period, cells were fixed. Focal adhesion complexes were visualized by immunofluorescence staining of vinculin (green), as described in Materials and Method. Actin stress fibers were detected with rhodamine-phaloidin (red). A. Representative fields from three independent experiments in PLC are shown. B. Focal adhesion complexes in each cell were counted. All results are expressed as a ratio to control (CAT) cDNA transfected PLC (CAT; clear bars, twist; black bars). The data are the mean ± SEM of at least three independent studies each performed in triplicate. Statistical analysis was performed by Student's *t*-test: **P < 0.01 (vs CAT cDNA transfected cells).

To elucidate the mechanisms involved in increased migration, we assessed the morphology of twist expressing cells. We utilized immunofluorescence double staining for actin cytoskeleton and focal adhesion to examine whether twist expression mediates a change in cell shape, stress fiber organization, and/or focal adhesion. Phase-contrast microscopy (data not shown) and immunofluorescence staining demonstrated that twist induced transformation from polarized, tight contact, rectangular-shape cell morphology to scattered, asteroid or fibroblast-like cell morphology. Twist expressing cells had reduced actin filament (Figure 5A). Vinculin staining demonstrated that twist expression markedly reduced vinculin localization with respect to focal contact in PLC cells (Figure 5A). Quantitative analysis of focal adhesion revealed that twist expression diminished focal adhesion by 52 ± 7%, 61 ± 6%, and 52 ± 5% in PLC, HepG2, and Huh7 cells, respectively (Figure 5B).

### siRNA against twist reduced cell migration activity in twist-positive HCC cell lines

**Figure 6:**
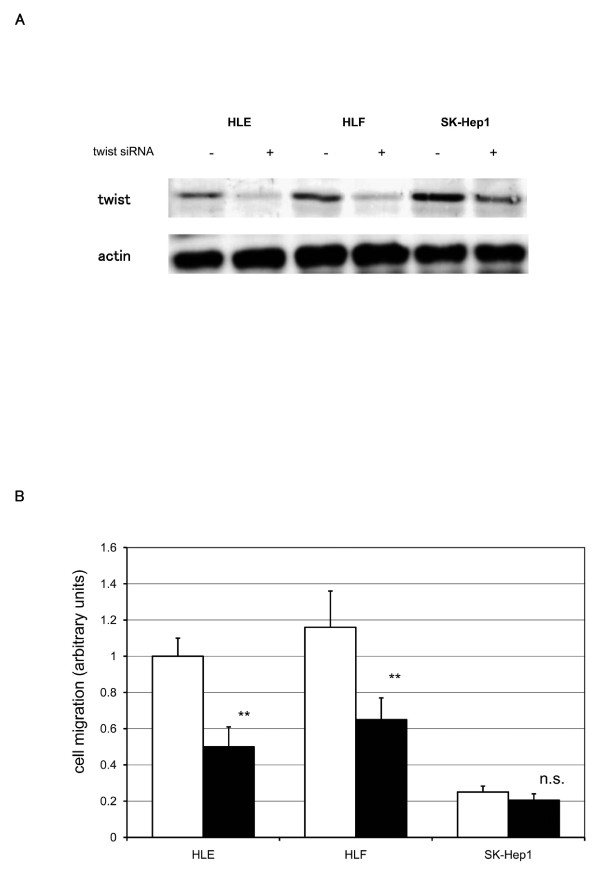
**Effect of gene silencing using twist siRNA on cell migration**. A. After treatment with twist siRNA duplexes, twist protein expression was analyzed by SDS-PAGE and immunoblot using anti-twist antibody. Immunoblotting for α-actin expression levels was used to verify equal loading of cellular protein. Shown are representative blots of more than three independent studies. B. After treatment with twist siRNA duplexes, cell migration assay was performed, as described in Materials and Methods. All results are expressed as a ratio to control siRNA treated HLE (control siRNA; clear bars, twist siRNA; black bars). The data are mean ± SEM of at least three independent studies, each performed in triplicate. Statistical analysis was performed by Student's *t*-test: *P < 0.01; n.s., not significant (vs control siRNA treated cells).

To examine the effect of twist suppression in twist-positive HCC cell lines, siRNA was used. siRNA blocked twist expression in twist-positive HCC cell lines (Figure 6A). Twist siRNA inhibited cell migration by 50 ± 11%, 44 ± 10%, and 18 ± 14% in HLE, HLF, and SK-Hep1, respectively (Figure 6B).

## Discussion

In the current study, we demonstrate that twist induces pro-migratory effects in HCC cell lines by inducing EMT.

Cancers such as HCC are multi-step processes associated with changes in gene expressions involved in cell proliferation, invasion, and metastasis. With recent advances in ablation techniques and hepatic resection, there have been marked improvements in local tumor control for HCC treatment [7,8]; however, patients with metastatic HCC still have a poor prognosis. Management of metastasis will therefore contribute to the improvement of prognosis for the HCC patient.

Various metastasis-related genes have been reported for HCC and other cancers. Twist, a transcription factor of the basic helix-loop-helix class, was identified as one of the most up-regulated genes in a metastatic murine breast tumor by microarray analysis [24]. Twist was originally reported as a master regulator of embryonic morphogenesis [31-33]. Recent studies revealed that twist-induced EMT enhances tumor metastasis [24,34]. It is reported that twist expression is elevated in invasive lobular carcinoma in human breast cancer [35]. Another study demonstrated up-regulation of twist in a diffuse-type gastric carcinoma in contrast to a well-differentiated intestinal carcinoma [36].

We first assessed twist expression in HCC cell lines and HCC tissues (Figures 1, 2A, B and Table 1) and found that some of these cell lines and tissues expressed twist protein or mRNA. In HCC tissue samples expressing twist, there were no significant clinicopathologic finding (Table 1). The HCC tissues were collected from HCC cases that could be operated on; thus, no case had distant metastasis. Immunohistochemical observations revealed that twist expression occurred in both cytoplasm and nuclei of HCC tissues. It is reported that high-level nuclear twist expression is associated with distant metastasis of other cancers [37]. In most HCC tissue samples, twist was expressed predominantly in the cytoplasm, probably because these cases did not have distant metastasis. Further studies with more cases, including those with distant metastasis, are necessary to evaluate the relationship between twist expression and clinical features of HCC. Twist expression in other carcinomas correlates with HCC metastasis [26]. In this report, the researchers focused on EMT and a decrease in E-cadherin expression caused by twist expression. E-cadherin is a key regulatory molecule involved in cell-cell adhesion. In the current study, we focused on the cell-substratum contact that regulates cell migration with respect to metastasis.

We classified six HCC cell lines into two groups: twist-positive and twist-negative (Figures 2A, B). In accordance with previous reports, twist expressing cells were classified in nondifferentiated carcinoma cell lines [38-42]. Morphologic observation also showed that twist expressing cells were nondifferentiated or had mesenchymal-like morphology (data not shown).

Cell proliferation and migration activities were assessed and compared between the two groups of HCC cell lines (Figures 2C, D). Twist-positive HCC cell lines showed higher cell migration activity compared with twist-negative cell lines. On the other hand, no difference was observed in cell proliferation activity of the twist-positive and twist negative HCC cell lines.

To elucidate the effect of twist expression in HCC cells, twist cDNA was introduced into twist-negative HCC cell lines. Ectopic expression of the *twist *gene successfully induced twist protein expression in twist-negative cell lines (Figure 3A). Twist expression did not increase cell proliferation in these cells (Figure 3B); however, cell migration activities increased in the three cell lines (Figure 3C). In agreement with a former report [26], these results demonstrated that twist expression may regulate cell migration in HCC.

Previous research has demonstrated that there are similarities between metastatic cancer cells and embryonic stem cells [43]. In epithelial cells, high expression of twist is associated with a loss in epithelial markers, including E-cadherin and β-catenin [24]. Instead, these cells express mesenchymal markers, including vimentin and N-cadherin. Because E-cadherin is essential for epithelial cell-cell adhesion, loss of E-cadherin expression would likely promote cancer metastasis. EMT causes aggressive behavior in HCC cell lines. Consistent with other reports, ectopic twist expression resulted in EMT in HCC cell lines (Figure 4).

Morphologic observations of HCC cell lines showed epithelial-like, well-differentiated carcinoma, as in PLC (Figure 5A), HepG2, and Huh7 (data not shown); these are all twist-negative HCC cell lines. On the other hand, undifferentiated carcinomas, such as HLE, HLF, SK-Hep1, are twist-positive HCC cell lines. Ectopic introduction of twist converted the characteristics of well-differentiated HCC cell lines to resemble those of undifferentiated cell types. Observing the morphology of cells with ectopic twist expression, we hypothesized that cell-substratum contact is reduced in these cells. Vinculin staining was utilized to visualize the focal adhesion contact (Figure 5A). In all, three twist-negative HCC cells, ectopic twist expression decreased the number of focal adhesion contacts by more than 50% (Figure 5B).

Focal adhesions are known to induce integrin signaling, which activate cell migration. Integrin signaling pathway modulates the activity of Rho family GTPases, such as cdc42, Rac1, and RhoA [44]. These proteins are key regulators of the actin cytoskeleton, which is involved in dynamic cellular activity. Cdc42 and Rac1 stimulate the formation of two types of membrane protrusions, known as filopodia and lamellipodia [44,45]. RhoA activation leads to the formation of focal adhesion and contractile bundles of actin and myosin, known as stress fibers [44], and increases levels of vinculin presenting focal adhesions [46]. In contrast, a decrease in RhoA activation may explain our observed decrease in cytoskeletal F-actin and vinculin expression observed in twist-induced HCC cell lines. In the current study, we did not investigate the mechanisms of inactivation of RhoA protein through overexpression of twist.

To evaluate the role of twist expression in regulating cell migration in HCC cell lines, we used twist siRNA. Twist-positive HCC cell lines were treated with twist siRNA. Twist siRNA successfully blocked the twist expression in twist-positive HCC cell lines (Figure 6A). Blocking of endogenous twist resulted in a decrease in cell migration related activities of HCC cell lines (Figure 6B). These results emphasize that twist regulates cell migration in HCC cells.

## Conclusion

In the current study, we demonstrated that twist expression regulates cell migration in HCC by decreasing cell-substratum adhesion. However, further studies are required to elucidate the mechanisms of decrease in focal adhesion. Twist and related molecules could be a possible therapeutic target for metastatic HCC.

## Competing interests

The authors declare that they have no competing interests.

## Authors' contributions

HS conceived the study design and drafted the manuscript. NM performed the experiments with assistance from NT, NU, ST, SN, MU, and AT. SN, YK, KN, and TY assisted in collection of HCC tissues. YN performed the immunohistochemical studies. KY provided financial support and participated in the analysis and discussion of results. All authors read and approved the final manuscript.

## Pre-publication history

The pre-publication history for this paper can be accessed here:

http://www.biomedcentral.com/1471-2407/9/240/prepub
